# The Other Side of the Shield

**Published:** 1891-02-28

**Authors:** 


### The Doctor's Armchair.

### THE OTHER SIDE OF THE SHIELD.

On Friday week representatives of the medical profession gathered together from all parts of the country, but chiefly from the "cold and moral North," to lay their views before Her Majesty's Most Honourable Privy Council on the subject of the Midwives' Registration Bill. One of the most eager opponents of the Bill, an old and valued friend of the writer's, who travelled two hundred miles to table his protest, called at the editorial sanctum, and freely expounded his views by the "space of one hour." A newspaper, like a judge, need claim no credit for opening its columns to the supporters of both sides of any particular contention. No newspaper, indeed, deserves to be read, or even to continue to live, which stands for anything else but freedom and fair play all round. Last week we discussed the Registration of Midwives from the point of view of poor women who employ midwives; but from the point of view, as well, of a very large portion of the medical profession. In the present article an attempt will be made to state and consider some of the objections which a certain section of the medical profession entertain towards the Bill.

It is affirmed, in the first place, that if midwives be registered, fifteen thousand women, most of whom are destitute of even the elements of a liberal education, and all of whom are entirely ignorant of the science and art of general medicine, will be added to the Medical Register. Not only will fifteen thousand of this class be converted into recognised midwifery practitioners by a stroke of the State pen, but a State door will be opened whereby fifteen or even fifty thousand additional female practitioners may be added to the Register, none of whom need necessarily be persons of liberal education, and none of whom will possess any knowledge whatever of general medicine; and yet all of whom will be registered and licensed to practise one branch of the medical art; and all of whom may, possibly, by virtue of their licence, claim to practise many other branches of that art.

Now this is a contention, the force of which we cannot deny. It is true that if midwives be registered some fifteen thousand persons will be immediately added to the ranks of medical practitioners. It is also true that the registration and State recognition of a midwife's position may encourage large numbers of more educated women to enter the midwife's profession. It is also the fact that any person who has placed a single foot on the highway of medical practice is strongly disposed to claim a "right of way" over the whole road. That this is so is proved by the attitude of consulting chemists and unqualified practitioners, as well as by that of some trained nurses and midwives. There is undoubtedly impending a serious change in the conditions of general medical practice. This is as evident to the observant eye as are the lengthening days of January and February or the first signs of spring in early March.

But the reasonable doctor has to ask himself quite a series of questions before he either gives way to the frantic imbecility of despair, or rises up in the stupid and unreasoning obstinacy of a futile opposition. He will ask himself, naturally and inevitably, whether the changes he fears are likely to affect him, financially or otherwise, for the worse? But he must also ask himself other questions; and an initial one, which, like certain algebraic formulæ, would enable him to answer many others, is this: Do doctors exist for the public, or does the public exist for doctors? If the public exists for doctors, then the interests of the public must give way to those of doctors; and whenever a change is threatened, a sufficient reason for putting a veto on the change will be that it will be injurious to the interests of the medical profession. Can it be the case, however, that there is a single man in the whole profession who is so blinded by self-interest as to take up an attitude of this kind? We do not believe it, even of a single man! To believe it of the whole profession would be to hold the conviction that it was a profession of madmen.

The rational medical man will further ask himself such questions as these: Do the best interests of poor women—that is, of the public—demand that competent knowledge and skill shall be available for them at the time of parturition? And if public opinion is determined that poor women not only ought to have, but shall have, competent knowledge and skill at that time, has the medical profession power enough to resist the force of that opinion; and is it wise to make the attempt to resist it? Medical men have not the power to resist public opinion, and to obstruct its State action; and they certainly will not be wise to make the attempt. It is to be borne in mind that the medical profession, like the clerical and the legal, holds a kind of privileged position by State licence, and is protected to a certain extent from open and unlimited competition. But this very fact makes the profession more or less obnoxious to certain influential minds; and any action taken by medical men to conserve their own special advantages at the expense of the public invariably rouses up angry and hostile feelings, which spread from the few to the many, until the whole profession becomes an object of public odium. Those doctors who bring about such a state of public feeling as this are not the friends of the medical profession, but its enemies.

The doctors who are opposing the Midwives' Registration Bill have succeeded in persuading themselves that self-interest has nothing to do with their action, but that one of their chief motives is a regard for the safety and well-being of poor parturient women. Now there was in olden times a very prudent man of the name of Paul, to-day called Saint Paul; and this shrewd Jew said, "If we would judge ourselves we should not be judged." Let the medical man ask himself this question: Why am I so tremendously active and anxious on behalf of poor lying-in women in the month of February, 1891, when I have never shown the least activity and anxiety on their behalf in my whole life before? If doctors will ask themselves this question they will prevent the public asking it. But if they do not, the public will certainly ask it, and they will answer it in only one way. They will say: You doctors do not care one straw for poor lying-in women, except as they affect your own self-interest; and the proof of this lies in the fact that you have never, in the hundreds of years of your professional history, taken a single disinterested step to promote the safety and comfort of those women in their time of need. This, of course, would not be quite true, but it would be true enough for the purposes of those laymen who are always ready to pillory the medical profession on the slighest conceivable occasion.

There is much more which might be said on both sides of this controversy, but space forbids. In our judgment the interests of the poor women should first be thought of, and those interests will be first thought of by the public and the powers that be. But we feel convinced that the true interests of the doctors lie in promoting the education and registration of midwives, and not in opposing them. The present policy of a portion of the profession is a policy of suicide, and of suicide accompanied by public odium. Let no man who respects himself and his profession persist in such a policy without giving much more time and deeper consideration to the subject than he has yet bestowed upon it.

